# Effects of Genotype, Storage Temperature and Time on Quality and Compositional Traits of Cherry Tomato

**DOI:** 10.3390/foods9121729

**Published:** 2020-11-25

**Authors:** Miriam Distefano, Elena Arena, Rosario Paolo Mauro, Selina Brighina, Cherubino Leonardi, Biagio Fallico, Francesco Giuffrida

**Affiliations:** Dipartimento di Agricoltura, Alimentazione e Ambiente (Di3A), University of Catania, 95123 Catania, Italy; miriam.distefano@unict.it (M.D.); earena@unict.it (E.A.); selina.brighina@unict.it (S.B.); cherubino.leonardi@unict.it (C.L.); biagio.fallico@unict.it (B.F.); francesco.giuffrida@unict.it (F.G.)

**Keywords:** *Solanum lycopersicum* L., refrigerated storage, fruit quality, carotenoids, total polyphenols content

## Abstract

The experiment addressed the effects of two storage temperatures, namely 10 (T_10_) and 20 °C (T_20_), on main quality and functional traits of three cherry tomato cultivars (‘Eletta’, ‘Sugarland’ and ‘Ottymo’), after 0 (S_0_), 7 (S_7_) and 14 (S_14_) days of storage. At T_10_ both fruit weight and firmness were better retained during storage. At S_14_, T_10_ promoted fruit Chroma and overall fruit color deviation (ΔE*_ab_). Total polyphenols content (TPC) of fruits peaked at S_7_ (4660 mg GAE kg^−1^ DW) then declined at S_14_ (by 16%), with the highest values recorded at T_10_. Lycopene showed a similar trend, but with a higher average concentration recorded at T_20_ (488 mg kg^−1^ DW). β-carotene content peaked at S_14_, irrespective of the storage temperature. At S_14_, the concentrations of phytoene and phytofluene were higher at T_20_ (48.3 and 40.9 mg kg^−1^ DW, respectively), but the opposite was found at S_7_. ‘Sugarland’ and ‘Ottymo’ showed the highest ΔE*_ab_ along storage, with the former cultivar proving the highest TPC and lycopene content, whereas ‘Eletta’ did so for phytoene and phytofluene. Our results suggest that unravelling the possible functional interactions among these three carotenoids would allow for a better orientation of breeding programs, targeting the phytochemical evolution of tomatoes during refrigerated storage.

## 1. Introduction

Tomato (*Solanum lycopersicum* L.) is one of most important vegetable crops throughout the world, with an estimated production of 182 Mt from more than 4.8 Mha cropland [1]. In the Mediterranean basin it is the primary field and greenhouse vegetable crop [2], since tomato strongly characterizes the Mediterranean diet, hence its consumption is widely spread around this macroarea [3]. Fresh tomatoes commercialization is often characterized by significant temporal gaps among production and consumption. This implies the optimization of quality maintenance of the product along the distribution chain, in order to match the consumers’ sensorial and nutritional demands [4]. Indeed, fresh vegetables are perishable commodities, whose postharvest decay represents a primary matter of social concern in terms of economic (loss of capital, fuel and manpower) and environmental costs (due to landfilling), associated to losses of valuable phytonutrients [5]. In this context, temperature is a key factor to extend quality of fresh horticultural products along the distribution chain [6]. Because of its sensitivity to chilling injuries [7], the optimization of tomatoes cold storage implies a compromise between temperatures low enough to slow down the ripening process but high enough to generate either no or tolerable side effects on the main organoleptic and nutritional traits [8]. Similarly to other plant foods [9], tomato is a source of many valuable phytonutrients having potential health benefits, including minerals, vitamins C and E, organic acids, polyphenols and carotenoids [10]. Carotenoids represent by far the most studied phytochemical fraction of tomatoes [11], which are considered the main dietary source of lycopene [12], i.e., the prevailing constituent conferring the typical pigmentation to red-ripe fruits. From a nutritional viewpoint, lycopene is a powerful antioxidant, whose intake has been linked to reduced frequency and severity of several types of cancer and heart diseases [13]. Moreover, it has been indicated as the most effective singlet oxygen quencher among all known carotenoids [14]. β-carotene is the second main carotenoid constituent of tomato fruits [15]. It is a red-orange pigment having strong chemoprotective functions and the highest provitamin A activity in the human metabolism, and its deficiency can result in xerophthalmia, blindness, and even premature death [16]. Although both carotenoids can be specifically ingested through dietary supplements, scientific evidences seem to point out stronger health benefits associated to their direct assumption from tomato matrices, likely as a consequence of synergistic effects involving other naturally occurring compounds [17]. Among these, the colorless carotenoids phytoene and phytofluene have been supposed to have biological activity, as in the case of skin protection from UV-induced erythema or in the protection of human lipoproteins from oxidation [18].

Over the last decades, cherry tomato has been intensively targeted in breeding programs of many seed companies, in order to match the evolving standards in tomato production, commercialization and consumption [19]. Consequently, the currently available cultivars are characterized by better functional profile than the past [20], wide compositional variability [21] and rapid temporal turnovers. Such diversification and dynamism represent a challenging task to optimize the product management along the distribution chain, since postharvest quality modifications are strongly affected by both storage conditions and genotype [22]. Hence, to address the growing demands for tomatoes with high quality and functional profiles, it is appropriate to in-depth the knowledge about whole patterns of change in these properties, as a function of the storage conditions applied to the emerging germplasm.

Due to this, the aim of the present work was to investigate the postharvest modifications on main quality variables of three recently widespread cherry tomato cultivars in a Mediterranean environment induced by different thermal regimes (10 and 20 °C) and storage time (up to 14 days).

## 2. Materials and Methods

### 2.1. Experimental Site and Plant Material

A greenhouse experiment was carried out from February to June 2019, at the experimental farm of the University of Catania (Sicily, South Italy: 37°24′27″ N, 15°03′36″ E, 6 m a.s.l.). The climate of the area is semi-arid Mediterranean, with mild winters and hot, dry summers. An 800 m^2^, multi-aisle cold greenhouse was used, having a steel tubular structure with adjustable windows on the roof and along the sides, and covered with polycarbonate slabs. Three cherry tomato cultivars, namely ‘Eletta’, ‘Ottymo’ and ‘Sugarland’, recently diffused in the reference area, were grown in the experiment, chosen on the basis of their different main carpometric traits (Table 1). To this end, data were previously acquired from different local farms operating in comparable growth conditions.

### 2.2. Growth Conditions, Fruit Sampling and Storage

Plants were transplanted on 11th February 2019 within the greenhouse at the stage of two true leaves, in an open soilless cultivation system using 5 L plastic pots (20 cm height, 19 cm width), with perlite as growing medium (particle size 2–6 mm). Before transplanting, plantlets were selected for uniform size and health appearance, whereas pots were arranged in simple rows, adopting a 0.40 × 1.00 m rectangular format (center to center) and 1 plant per pot (2.5 plants m^−2^). Plants were grown at single stem up to the 8th cluster, whereas all clusters were pruned leaving 12 fruits, whose setting was allowed by using bumblebee hives. Each net experimental unit contained 12 plants. During the cycle, the crop was uniformly fertigated with a standard nutrient solution [23], adopting a leaching fraction of at least 75%, to avoid root zone salinization [24].

From 14 to 16 May, tomatoes belonging to the 4th cluster were harvested by hand at the red stage (stage F) according to Gautier et al. [25]. This was done to allow tomatoes to reach stage G (deep red) during postharvest, as it is usual among local growers. Soon after harvest, fruits were transported to the laboratory and processed for further analysis. Overall, 72 clusters were collected (8 clusters × 3 cultivars × 3 replicates) and divided in 3 batches for the characterization of fruits after 0 (harvest date), 7 and 14 days of storage (hereafter S_0_, S_7_ and S_14_, respectively), either at 10 ± 0.5 (T_10_) or 20 ± 0.5 °C (T_20_) and 85% relative humidity (RH). The lowest thermal regime was chosen since it represents a mild stressing conditions frequently adopted during transportation and storage of cherry tomatoes, whereas T_20_ was comparatively chosen as it simulates storage at room temperature [26]. Before storage, fruits were detached from rachis, selected for absence of defeats and uniform appearance within each genotype, washed with deionized water and dried with paper for further analysis. Fifteen to twenty-two fruits per replicate were placed in common commercial trays, i.e., transparent PET trays Mod. C500/41p (190 × 115 × 41 mm) covered with a perforated PET LC32 lid (Carton Pack s.p.a., Rutigliano, Italy) for a final net weight of 250 ± 8 g, then stored at the abovementioned conditions.

### 2.3. Carpometric Determinations

At each time point, fruit fresh weight was determined on 10 fruits per tray, then their firmness was determined through a Digital Texture Analyser mod. TA-XT2 (Stable Micro Systems, Godalming, UK) and defined as the force (N) needed to impress a 2 mm fruit deformation along the polar axis, between two steel plates.

### 2.4. Cherry Tomato Quality Variables

For each sample, ~50 g of cherry tomato were homogenized up to a puree in a home blender (La Moulinette, Groupe SEB, Écully, France) and immediately analyzed for: soluble solids content, dry matter, pH, total acidity (TA), reducing sugars, total polyphenols and carotenoids profile and content.

The soluble solids content (SSC) was estimated with an Abbe refractometer 16531 (Carl Zeiss, Oberkochen, Germany) at 20 °C and the results were expressed as °Brix. The dry matter was determined by gravimetric analysis. An aliquot of cherry tomato puree were placed in an oven at 70 °C (Thermo Fisher Scientific, Waltham, MA, USA) until the constant weight [27]. The pH was measured using a pHmeter (Mettler Toledo, MP 220), and tritatable acidity (TA) was determined by titrating an aliquot of the puree sample with 0.05 N NaOH to pH 8.1. TA was expressed as g kg^−1^ of cherry tomato fresh weight (FW), as citric acid.

Reducing sugars (fructose and glucose), were estimated using Fehling’s method according to the official Italian method of analysis (D.M. 3.2.1989, GU n.168/1989). An aliquot of the puree sample (20 g) were transferred into a volumetric flask (50 mL) and neutralized with 1 N NaOH. Subsequently sample was cleared by the addition of 10 mL saturated sodium sulphate decahydrate and 5 mL saturated basic lead acetate. The samples were diluted up to 50 g with distilled water, mixed and centrifuged for 10 min at 10,000 rpm. The supernatant was filtered through a filter paper (Whatman No 1, Whatman International, Maidstone, UK) and used to completely reduce in hot condition a mixed of the Fehling’s solution using methylene blue solution as indicator. The Fehling solution was prepared as follow: 5 mL of each stock Fehling solution A and B were mixed with 40 mL of distilled water immediately before the determination. Results were expressed as g of reducing sugars kg^−1^ of dry weight (DW) and all analysis were conducted in triplicate.

### 2.5. Fruit Chromatic Coordinates

The fruit chromatic coordinates were measured as described by McGuire [28] on the equatorial axis of whole fruits (two measurements per fruits), through a tristimulus Minolta Chroma meter (model CR-200, Minolta Corp.) calibrated with a standard white tile (UE certificated) with illuminant D65/10°, measuring color in terms of lightness (L*), green-red axis (a*) and blue-yellow axis (b*). Fruit color was described as (a*/b*)^2^, Chroma [as (a*^2^ + b*^2^)^1/2^], tomato color index [TCI = 2000 a*/L*(a*^2^ + b*^2^)^1/2^] and total color difference [ΔE*_ab_ = (ΔL*^2^ + Δa*^2^ + Δb*^2^)^1/2^], this last describing the color deviation recoded at S_7_ and S_14_.

### 2.6. Total Polyphenols Content

The extraction of polyphenol compounds was performed according to Atanasova et al. [29] with some modifications. An aliquot of cherry tomato puree sample (1 g) was mixed and shacked with 40 mL of acetone (80% solution in distilled water) and left in the dark, overnight at room temperature. After that, each sample was filtered (0.45 µm Albet) and the supernatant was collected for determination of total polyphenols content (TPC). This was determined according to Gahler et al. [30] using the Folin-Ciocâlteu reagent and measuring spectrophotometrically the absorbance at 725 nm using a Perkin Elmer lambda 25 Uv-Vis spectrometer. Gallic acid was used as standard (standard curve, 0.29–8.18 mg kg^−1^; *R*^2^ = 1.00) and TPC was expressed as mg gallic acid equivalents (GAE) kg^−1^ on a dry weight (DW) basis. All analyses were carried out in triplicate.

### 2.7. Carotenoids Extraction and HPLC Analysis

Tomato carotenoids were extracted using the method of Siracusa et al. [31]. An aliquot of the cherry tomato puree sample (0.5 g) was transferred into a vial and 5 mL of a n-hexane/acetone/ethanol (2:1:1) solution were added. The vial was left shaking for 40 min. in the dark at room temperature. Subsequently 1 mL of H_2_O (HPLC grade) was added and a further 2 min. agitation was applied. The resulting heterogeneous mixtures were left decanting until phases separation. The apolar coloured layers were transferred into an amber vial and analysed.

Quantitative analyses were carried out on an HPLC (Shimadzu USA Manufacturing Company Inc., Class VPLC-10 Dvp, Canby, OR, USA) equipped with a DAD (Shimadzu SPD-M10Avp). The column was a Gemini NX C18 (150 × 4.6 mm; 3 μm particle size; Phenomenex, Italy), fitted with a guard cartridge packed with the same stationary phase. The flow rate was 0.7 mL/min. and the injector volume was 20 μL. Carotenoids were eluted with the following gradient of A (Methanol: H_2_O 75:25) and B (Ethyl acetate): T0 30% B; T15 82% B; T25 30% B. All reagents used were HPLC purity grade: water, methanol and Ethyl acetate were obtained from Merck. The wavelength range was 220–660 nm, and the chromatograms were monitored at 473 nm for lycopene; at 453 nm for β-carotene; at 348 nm for phytofluene and at 288 nm for phytoene. Carotenoids were identified by splitting the peak of the carotenoids from the tomato-solution sample with a standard of β-carotene and lycopene; (*p* ≥ 95% and *p* ≥ 98%, Sigma-Aldrich, St. Louis, MO, USA) and by comparing retention times and UV spectra with those of standards. Quantification of β-carotene and lycopene was performed using external calibration curves; for phytofluene and phytoene the calibration curve of β-carotene was used. Linearity was checked for β-carotene between 3.36 and 21 mg kg^−1^ (*R*^2^ = 1.00) and lycopene between 2.56 and 40.0 mg kg^−1^ (*R*^2^ = 1.00). All analyses were performer in triplicate, including the extraction procedure, and the results were expressed as mg kg^−1^ DW.

### 2.8. Statistical Procedures

All data were subjected to Shapiro-Wilk and Levene’s test, in order to check for normality and homoscedasticity, respectively, then to a factorial ‘storage temperature × genotype × storage time’ (T × G × S) analysis of variance (ANOVA), according to the experimental layout adopted in the experiment. Percentage data were Bliss transformed before the ANOVA (untransformed data are reported and discussed), whereas multiple means comparisons were performed through Tukey’s honestly significant difference (HSD) test (*p* ≤ 0.05). All calculations were performed using Excel version 2016 (Microsoft Corporation, Redmond, WA, USA) and Minitab version 16.1.1 (Minitab Inc., State College, PA, USA).

## 3. Results

**Figure 1:**
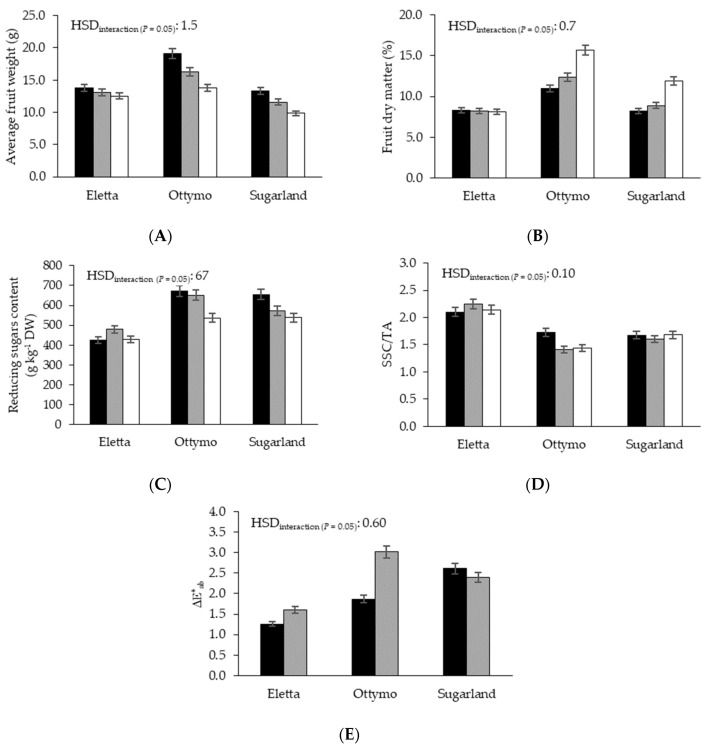
Average fruit weight (**A**), fruit dry matter (**B**), reducing sugars content (**C**), SSC/TA (**D**) and ΔE*_ab_ (**E**) as affected by ‘genotype × storage time’ interaction. Black bars: S_0_; grey bars: S_7_; white bars S_14_.

**Figure 2:**
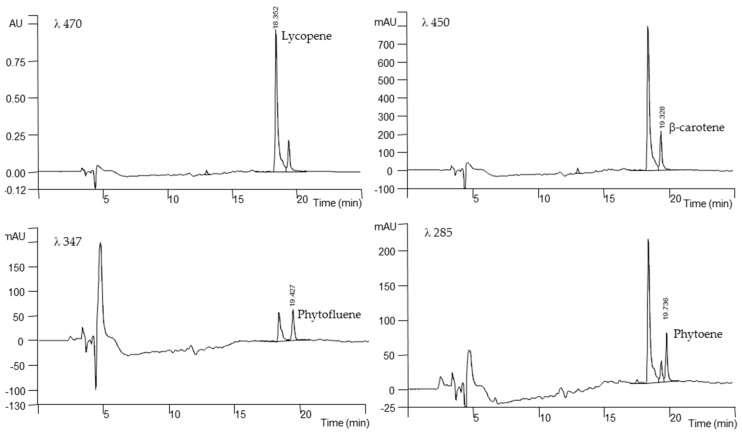
HPLC profile of carotenoids extracted from cherry tomato ‘Sugarland’ at harvest date (S_0_).

**Figure 3:**
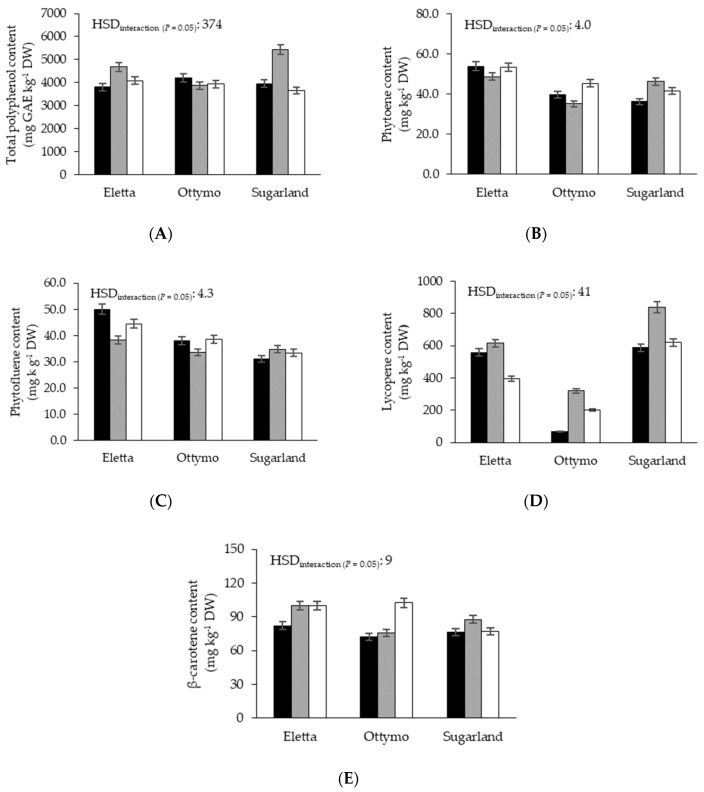
Total polyphenols (**A**), phytoene (**B**), phytofluene (**C**), lycopene (**D**) and β-carotene (**E**) content as affected by ‘genotype × storage time’ interaction. Black bars: S_0_; grey bars: S_7_; white bars S_14_.

In the present study, the significance resulting from the ANOVA related to storage temperature (T), genotype (G) and storage time (S) and their first order interactions is reported in Table 2 (Fisher-Snedecor *F*-test), whereas their effects on variable means are reported in Table 3, Table 4, Table 5 and Table 6 and Figure 1, Figure 2 and Figure 3.

### 3.1. Carpometric Traits

Average fruit weight showed a significant ‘T × G’ interaction since, passing from T_10_ to T_20_, ‘Ottymo’ and ‘Sugarland’ showed the highest reduction (−9%, on average) (Table 3). Moreover, both cultivars proved the highest decline of fruit weight at the end of the storage period, as this variable was reduced by 28%, on the average of both cultivars (Figure 1A).

Fruit dry matter, proved a higher value at T_20_ than at T_10_, reaching the highest rise among the thermal regimes in ‘Ottymo’ (+15%) and ‘Sugarland’ (+12%) (Table 3). Both genotypes highlighted the highest rise during the storage period, as their fruit dry matter increased by 44% on average, passing from S_0_ to S_14_ (Figure 1B). Differently, at T_20_ fruit firmness was significantly reduced, with ‘Ottymo’ showing the strongest decline passing from T_10_ to T_20_ (−19%) (Table 3). For this variable, a decreasing trend was recorded along the storage period, since, by comparison with the initial value, fruit firmness was reduced by 22% at S_14_ (Table 3).

### 3.2. Cherry Tomato Quality Variables

On the average of the other factors, ‘Ottymo’ and ‘Sugarland’ proved the highest reducing sugars content (581 g kg^−1^ DW, on average), whereas the former cultivar proved the lowest pH; differently, ‘Sugarland’ minimized the SSC/TA ratio (Table 4). Both reducing sugars content and SSC/TA declined passing from S_0_ to S_14_ (by 14 and 4%, respectively). For the former variable, the strongest reduction along the storage period was noticed in ‘Ottymo’ and ‘Sugarland’ (−19%, on average) (Figure 1C), whereas for SSC/TA the only significant reduction within the S_0_–S_14_ period was found in ‘Ottymo’ (−17%) (Figure 1D).

### 3.3. Chromatic Variables

Among the chromatic variables, Chroma and ΔE*_ab_ showed a similar response to storage temperature, as they were both increased at T_10_ (by 4% and 27%, respectively) (Table 5). For Chroma, the increase under cold storage was particularly evident passing from S_7_ (24.1) to S_14_ (26.0, +8%) (Table 5). Among the studied genotypes, ‘Eletta’ showed the highest (a*/b*)^2^ and Chroma (0.81 and 26.4, respectively) and the lowest ΔE*_ab_ (1.43), whereas the lowest TCI was found in ‘Sugarland’ (31.3) (Table 5). All the chromatic variables significantly increased between S_7_ and S_14_, but for ΔE*_ab_ such temporal rise was more prominent in ‘Ottymo’ (by 61%) (Figure 1E).

### 3.4. Total Polyphenols Content

Total polyphenols content (TPC) was significantly higher at T_10_ (4327 mg GAE kg^−1^ DW) that at T_20_ (4034 mg GAE kg^−1^ DW) (Table 6), but with strong interactive effects with genotype and storage time. Indeed, while ‘Eletta’ showed no differences among the 2 thermal regimes, TPC was strongly promoted by the lowest thermal regime in ‘Sugarland’ (+20%), followed by ‘Ottymo’ (+9%) (Table 6). As regards its temporal trend, TPC significantly increased passing from S_0_ to S_7_ (+17%) then sharply declined at S_14_ (−16%), with a steeper rise in the S_0_–S_7_ period recorded at T_10_ (+22%) than at T_20_ (+12%) (Table 6). Moreover, the studied genotypes displayed different time-courses of TPC along the storage period, since ‘Sugarland’ proved the highest TPC rise passing from S_0_ to S_7_ (+37%) followed by the strongest decline at S_14_ (−33%) (Figure 3A).

### 3.5. Carotenoids Content

Figure 2 shows the HPLC carotenoids profile extracted from cherry tomato ‘Sugarland’. At harvest date, the level of lycopene ranged from 68.1 to 582.5 mg kg^−1^ DW in ‘Ottymo’ and ‘Sugarland’, respectively, followed by β-carotene, which varied from 72.8 to 82.17 mg kg^−1^ DW, in ‘Ottymo and ‘Eletta’, respectively. Among the genotype tested, ‘Eletta’ proved the highest levels of both phytoene and phytofluene (54.2 and 50.7 mg kg^−1^ DW, respectively). The levels determined in ‘Sugarland’ and ‘Ottymo’ varied from 31.0 to 38.2 mg kg^−1^ DW, for phytoene and from 36.1 to 39.9 mg kg^−1^ DW, for phytofluene, respectively.

The phytoene content of the studied genotypes proved different time courses among the 2 thermal regimes, as it significantly increased passing from S_7_ to S_14_ when the T_20_ storage was considered (from 41.2 to 48.3 mg kg^−1^ DW, +14%) (Table 6). Among the genotypes, ‘Sugarland’ proved the highest phytoene rise passing from S_0_ to S_7_ (from 36.2 to 46.1 mg kg^−1^ DW, +28%), whereas in ‘Ottymo’ a significant increase was recorded between S_7_ (35.1 mg kg^−1^ DW) and S_14_ (45.3 mg kg^−1^ DW, +29%) (Figure 3B).

Regarding phytofluene, the lowest storage temperature showed a depressive effect in ‘Eletta’ (in which it was reduced by 9%) and the opposite in ‘Ottymo’ (in which it increased by 10%) (Table 6). Phytofluene content proved also wider temporal oscillations at T_20_, as the initial value was reduced by 6.4 mg kg^−1^ DW at S_7_ (−9%), then increased by 6.4 mg kg^−1^ DW at S_14_ (+19%) (Table 6). Such temporal oscillations proved to be genotype-dependent too, since ‘Eletta’ showed the highest reduction passing from S_0_ (50 mg kg^−1^ DW) to S_7_ (38.4 mg kg^−1^ DW, −23%), then the sharpest rise at S_14_ (44.5 mg kg^−1^ DW, +16%) (Figure 3C).

Lycopene was significantly affected by the storage temperature, as it was lower at T_10_ than at T_20_ (445 vs. 488 mg kg^−1^ DW), and this reduction was more marked for ‘Eletta’ (−12%) and ‘Sugarland’ (−6%) (Table 6). Moreover, T_20_ promoted a sharper lycopene rise than T_10_ passing from S_0_ to S_7_ (from 416 to 577 mg kg^−1^ DW, +39%) followed by a milder decrease at S_14_ (484 mg kg^−1^ DW, −16%) (Table 6). All the studied cultivars showed a significant decrease in lycopene content between S_7_ and S_14_ (ranging from 119 to 221 mg kg^−1^ DW in ‘Ottymo and ‘Eletta’, respectively), with ‘Ottymo’ and ‘Sugarland’ proving also a higher lycopene increase between S_0_ and S_7_ (by 252 mg kg^−1^ DW, on average) (Figure 3D).

β-carotene concentration proved to be not sensitive to the storage temperature, and was higher in ‘Eletta’ (94.0 mg kg^−1^ DW) than in the other genotypes (81.9 mg kg^−1^ DW, on average) and, over the storage period, increased up to 93.1 mg kg^−1^ DW at S_14_ (Table 6). However, such temporal increase was more marked in ‘Eletta’ within the S_0_–S_7_ period (from 82.1 to 100.0 mg kg^−1^ DW) and in ‘Ottymo’ in the S_7_–S_14_ one (from 75.9 to 102.3 mg kg^−1^ DW) (Figure 3E).

## 4. Discussion

The fruits stored at 10 °C showed a higher fruit weight and a lower dry matter content as compared to those stored at 20 °C, indicating that fruit transpiration and water loss were the main processes affected by storage temperature. As a consequence, at 20 °C tomatoes proved a higher loss of fruit firmness over time. The transpiration-driven softening of tomatoes during postharvest is a major problem, as it increases their susceptibility to damages along the distribution chain [32]. Moreover, fruit firmness is considered a key indicator of tomato freshness, able to influence the purchasing behavior of consumers [33]. However, despite cold storage is commonly practiced for reducing postharvest softening of tomatoes, the opposite effect can be found when too low storage temperatures are used, because of the tropical origin of the plant [8]. For this reason, storage temperature over 11–12 °C are advised for storing tomatoes, depending on fruit typology and ripening stage [32,33,34,35]. Nonetheless, the differences in terms of fruit weight and firmness we found among the 2 thermal regimes showed that storage at 10 °C was a suitable way to extend these main characteristics of tomato fruits. Among the studied cultivars, both ‘Sugarland’ (small-fruited) and ‘Ottymo’ (large-fruited) showed the highest fruit weight reduction during storage, consistent with their steeper rise in dry matter content. Differently, ‘Eletta’ (medium-fruited) proved the highest temporal stability in relation to both variables. Hence our results suggest that the genotypic attitude of cherry tomato to retain fruit weight and firmness during postharvest, is dependent from factors other than simply the fruit size (i.e., the ratio among berry volume and its external transpiring surface) [36], and likely due to the functional traits of the epicarp. Indeed, it has been reported that the dynamics of fruit water loss and consequent tissue collapse are influenced by genotypic differences in structural characteristics of the cuticle, whose alteration over time is an intrinsic feature of the genetically-programmed ripening process [37].

In tomato, the ethylene-driven ripening and senescence lead to the alteration of the carbon substrates content [38], as they are energy-requiring processes whose kinetic is influenced by the ambient temperature [24]. In our experiment, reducing sugars content, the ratio SSC/TA and fruit pH were not affected by the storage temperature, proving instead to be genotype-dependent. Despite their higher increase in dry matter, ‘Sugarland’ and ‘Ottymo’ highlighted the steepest drop in reducing sugars content at the end of storage period (by 19%, on average), denoting within the 10–20 °C range a temperature-insensitive acceleration of their autocatalytic metabolism. This demonstrates that no chilling disturbance in reducing sugars metabolism occurred in the experiment [34]. To this end, while the cultivars did not show appreciable pH variations during storage, ‘Ottymo’ proved the highest SSC/TA reduction over time, denoting its lowest suitability to keep unchanged the taste peculiarities. Indeed, the SSC/TA ratio is a pivotal organoleptic descriptor, as it is related to the overall balance in the perceived sweetness (SSC) and sourness (TA) of tomatoes [39].

Color is one of most important and widely used parameters to define the quality of tomato and tomato products [40]. When fresh tomato fruits are concerned, it is linked to fruit ripeness and firmness and is generally associated by consumers to tomatoes eating quality. In the present experiment, we used an array of chromatic variables summarizing the main color modifications occurring in tomato epicarp. Chroma, (a*/b*)^2^ and tomato color index have been related to quality traits of tomato [41,42], whereas ΔE*_ab_ has been successfully used to monitor the quality maintenance of potato sticks during refrigerated storage [43]. All these variables showed a certain variability among the studied cultivars, with two of them, namely Chroma and ΔE*_ab_, increasing at T_10_, overall indicating a higher deviation toward more vivid fruit colors. In particular, after 14 days of storage, a higher reduction of Chroma was recorded at 20 °C, a condition which matched the strongest decrease in fruit weight and firmness experienced by the studied cultivars. ‘Sugarland’ and ‘Ottymo’ proved higher ΔE*_ab_ variations during storage. According to Dattner and Bohn [44], independently from the deviation formula, two colors can be optically distinguished if Δ*E* > 1. The ΔE*_ab_ differences attained by ‘Sugarland’ and ‘Ottymo’ (2.47 units, on average) and ‘Eletta’ (1.43) indicate for the former cultivars a higher perceivable color deviation along the storage period, consistent with their higher qualitative decline in terms of fruit weight and turgor.

When phytochemical composition was concerned, total polyphenols, lycopene and β-carotene contents found in our experiment were substantially in line with those reported by Fernandes et al. [45] for cherry tomato ‘Moscatel RZ’ grown in hydroponic or semi-hydroponic systems. On the other hand, phytoene and phytofluene contents were very similar to those found in cherry tomato by Mapelli-Brahm et al. [46]. Plant polyphenols are a large group of phytochemicals involved in the regulation of plant growth, reproduction and response to the environmental stressors [47]. From a nutraceutical viewpoint, they have strong antioxidant properties probably implicated in the decreased incidence of cardiovascular diseases and certain forms of cancer [48]. Both thermal regimes promoted a bell-shaped postharvest trend of TPC, consisting in their sharp rise at S_7_, followed by a decrease at S_14_, this last indicating the onset of metabolic senescence processes [49]. However, such increase was more marked at 10 °C, suggesting the occurrence of a cold-adaptive response in up-regulating the polyphenols expression during postharvest storage. Indeed, several phenolic compounds typically accumulate in plant cells subjected to cold stress, as they contribute to the homeostasis of cold-induced reactive oxygen species (ROS) and to enhance the thickness of the cell wall, so preventing lipid peroxidation and cell collapse [47]. This would explain the best retention of fruit firmness recorded at 10 °C, indicating at the same time, the improvement of tomato phenolic profile as a benefit induced by a mild cold stress. Thus, although polyphenols have not been considered a priority target in tomato breeding programs, our results suggest that they could represent a sensitive target for improving the functional profile of the tomato, mostly during postharvest cold storage.

Regarding the carotenoid fraction, we recorded variable effects, resulting from different time-course response to storage temperature and duration. Lycopene displayed a bell-shaped temporal trend too since, under both storage temperatures, this carotenoid sharply increased at S_7_ then declined at S_14_. This trend substantially differed from that of β-carotene which continuously increased until S_14_, so confirming the higher stability of its postharvest accumulation in tomato [50]. According to Rodriguez-Amaya [51], carotenoids accumulation can continue during postharvest transport or storage, provided that the integrity of the fruit is maintained, so preserving the enzymatic activity responsible for carotenogenesis. Lycopene plays a paramount function in protecting the photosynthetic apparatus and plant lipid membranes, as its acyclic polyene structure (11 conjugated double bonds) increases its affinity for singlet oxygen and radical scavenging activity beyond the other carotenoids [52]. For this reason, it has been reported that oxidation is the main cause for lycopene degradation [14]. This could partly explain the depressive effect on lycopene content we recorded upon storing tomatoes in a stressing, ROS-inducing environment (10 °C). In this view, it is interesting to note the contrasting effect of cold storage on tomato compositional traits, resulting in a higher polyphenols accumulation in case of a lower lycopene content. This suggests the existence of a fine tuning among different classes of compounds in response to cold stress. However, by comparing the temporal trend of lycopene with that of its colorless precursors phytoene and phytofluene, clear time-dependent temperature effects on carotenogenesis where noticeable. Indeed, at S_7_, the highest lycopene content recorded at 20 °C matched the strongest reduction of both phytoene and phytofluene. In other words, the lowest the lycopene concentration the highest the accumulation of its precursors and vice versa. This implies that reduced transformation kinetics of both phytoene and phytofluene represented the earliest metabolic constraints recorded in response to the imposed cold stress. According to Dumas et al. [53] the over-expression of phytoene desaturase (leading to lycopene synthesis by desaturating both phytoene and phytofluene) is the most important upstream metabolic step in increasing the lycopene content of tomato fruits at harvest. Our results bear this out in postharvest conditions too, as they indicate that, under mild cold stress storage conditions, desaturation of phytoene and phytofluene represents the earliest metabolic bottleneck in lycopene synthesis of cherry tomatoes, hence a possible priority target to modulate the postharvest evolution of their nutraceutical profile. On the other hand, to which extent this implies a mid-term modification of the overall nutraceutical profile of tomato represents an interesting topic, taking into account that, despite they are not effective antiradicals as lycopene, phytoene and phytofluene are among the prevailing carotenoids found in human plasma and tissues, and their bioaccessibility following gastro-intestinal digestion of tomato juice has been found ~3–4 fold higher than that of lycopene [54,55].

Among the studied cultivars ‘Sugarland’ proved the highest lycopene and total polyphenols content, whereas ‘Eletta’ overcame the other cultivars for phytoene and phytofluene. Excepting β-carotene, which over time increased more sharply in ‘Eletta’ and ‘Ottymo’, these differences were still noticeable at the end of the storage period, regardless of the storage temperature. This highlights, beyond the environmental influence, the existence of a strong genetic component determining the stoichiometric relationships among lycopene and its precursors. Unravelling the possible interactive effects among these three carotenoids in generating the antioxidative health benefits [16,49] will allow for a better orientation of breeding programs toward the most convenient phytochemical evolution of tomatoes during refrigerated storage.

## 5. Conclusions

The results of the present experiment highlighted complex postharvest modifications of cherry tomatoes in response to the studied factors. By storing them under mild stressing conditions (10 °C) it was possible to improve the stability over time of carpometric traits (mainly fruit weight, firmness and Chroma) having commercial relevance, without alterations of compositional traits related to taste perception (reducing sugars content, SSC/TA and pH). Moreover, when compared to 20 °C, storing at 10 °C boosted the accumulation of total polyphenol and, at least in the short term (within 7 days of storage), the concentration of both phytoene and phytofluene, probably inhibiting their enzymatic desaturation leading to lycopene. This suggests their possible usefulness in modulating the nutraceutical evolution of cold stored cherry tomatoes during postharvest. This idea is reinforced by the stable varietal differences we found in terms of stoichiometric relationships among lycopene, phytoene and phytofluene. Regarding the varietal attitude to postharvest storage, the stability over time of fruit weight, dry matter content, SSC/TA and ΔE*_ab_ proved to be highly discriminant among cultivars, indicating the lowest ability of ‘Ottymo’ and ‘Eletta’ to maintain their fruit peculiarities over time. Thus, our results suggest the use of these variables to screen for cherry tomato germplasm suited to periods of postharvest storage.

## Figures and Tables

**Table 1:** Main information and fruit characteristics related to the cultivars selected for the study.

	‘Eletta’	‘Ottymo’	‘Sugarland’
Seed company	TSI Italia srl, Foggia (FG), Italy	Vilmorin Italia srl, Funo (BO), Italy	Rijk Zwaan Italia srl, Bologna (BO), Italy
Fruit color	Deep red	Red	Deep red
Average fruit diameter (mm)	15 ± 2	18 ± 2	12 ± 1
Average fruit weight (g)	15.0 ± 1.5	20.5 ± 2.5	12.0 ± 1.0

**Table 2:** *F*-test values of the main factors and their first order interactions related to observed variables, with the significance resulting from the analysis of variance.

Variable	Source of Variation					
	Storage Temperature	Genotype	Storage Time	(T) × (G)	(T) × (S)	(G) × (S)
Average fruit weight	13.6 ***	140.8 ***	65.6 ***	3.8 *	NS	8.1 ***
Fruit dry matter	10.6 **	88.4 ***	29.7 ***	3.5 *	NS	8.7 ***
Fruit firmness	8.7 **	29.4 ***	11.5 ***	3.9 *	NS	NS
Reducing sugars content	NS	50.2 ***	10.2 ***	NS	NS	3.8 *
SSC/TA	NS	265.2 ***	4.7 *	NS	NS	12.4 ***
Fruit pH	NS	19.6 ***	NS	NS	NS	NS
(a*/b*)^2^	NS	38.0 ***	6.3 **	NS	NS	NS
Chroma	39.4 ***	544.7 ***	30.9 ***	NS	13.2 ***	NS
Tomato color index	NS	39.9 ***	4.9 *	NS	NS	NS
ΔE*_ab_	8.1 **	15.1 ***	5.7 *	6.0 **	NS	5.0 *
Total polyphenols content	20.9 ***	9.5 ***	56.4 ***	17.2 ***	4.7*	32.5 ***
Phytoene content	NS	82.8 ***	7.5 **	NS	3.6 *	15.1 ***
Phytofluene content	NS	44.3 ***	6.5 **	6.3 **	3.9 *	6.8 ***
Lycopene content	33.8 ***	1462.3 ***	138.5 ***	3.6 *	9.8 ***	121.4 ***
β-carotene content	NS	17.4 ***	23.1 ***	NS	NS	11.7 ***

SSC: soluble solids content; TA: tritatable acidity. (T): storage temperature; (G): genotype; (S): storage time. NS: not significant; *, ** and ***: significant at *p* ≤ 0.05, 0.01 and 0.001, respectively.

**Table 3:** Carpometric variables of cherry tomato as affected by the main factors.

Variable		Genotype			Storage Time			Storage Temperature Mean
		‘Eletta’	‘Ottymo’	‘Sugarland’	S_0_	S_7_	S_14_	
Average fruit weight (g)	T_10_	13.3	17.2	12.0	15.3	14.1	12.9	14.1 ^a^
	T_20_	13.0	15.5	11.1	15.5	13.6	12.0	13.2 ^b^
	Mean	13.2 ^b^	16.4 ^a^	11.6 ^c^	15.4 ^a^	13.9 ^b^	12.5 ^c^	
HSD_interaction_			0.8			0.8		
Fruit dry matter content (%)	T_10_	8.2	12.1	9.1	9.1	9.4	10.9	9.8 ^b^
	T_20_	8.2	13.9	10.2	9.2	10.3	12.9	10.8 ^a^
	Mean	8.2 ^c^	13.0 ^a^	9.7 ^b^	9.2 ^b^	9.8 ^b^	11.9 ^a^	
HSD_interaction_			1.1			NS		
Fruit firmness (N)	T_10_	14.1	19.7	12.8	16.5	16.1	14.1	15.6 ^a^
	T_20_	13.6	16.0	11.6	16.6	12.9	11.7	13.7 ^b^
	Mean	13.8 ^b^	17.9 ^a^	12.2 ^c^	16.6 ^a^	14.5 ^b^	12.9 ^c^	
HSD_interaction_			2.2			NS		

Different letters among factor means indicate significance at Tukey’s HSD test (*p* ≤ 0.05). Interaction values (*p* = 0.05) related to ‘storage temperature × genotype’ and ‘storage temperature × storage time’ are reported. NS: not significant.

**Table 4:** Compositional variables related to fruit taste of cherry tomato as affected by the main factors.

Variable		Genotype			Storage Time			Storage Temperature Mean
		‘Eletta’	‘Ottymo’	‘Sugarland’	S_0_	S_7_	S_14_	
Reducing sugars content (g kg^−1^ DW)	T_10_	434	595	552	557	555	463	527 ^a^
	T_20_	420	597	579	565	536	499	529 ^a^
	Mean	427 ^b^	596 ^a^	565 ^a^	561 ^a^	545 ^a^	481 ^b^	
HSD_interaction_			NS			NS		
SSC/TA (adimensional)	T_10_	2.15	1.65	1.52	1.80	1.77	1.71	1.77 ^a^
	T_20_	2.18	1.66	1.54	1.86	1.74	1.80	1.79 ^a^
	Mean	2.16 ^a^	1.65 ^b^	1.53 ^c^	1.83 ^a^	1.76 ^b^	1.76 ^b^	
HSD_interaction_			NS			NS		
Fruit pH	T_10_	4.59	4.21	4.33	4.37	4.35	4.42	4.38 ^a^
	T_20_	4.61	4.22	4.38	4.38	4.42	4.42	4.40 ^a^
	Mean	4.60 ^a^	4.21 ^b^	4.36 ^ab^	4.37 ^a^	4.38 ^a^	4.42 ^a^	
HSD_interaction_			NS			NS		

Different letters among factor means indicate significance at Tukey’s HSD test (*p* ≤ 0.05). Interaction values (*p* = 0.05) related to ‘storage temperature × genotype’ and ‘storage temperature × storage time’ are reported. NS: not significant.

**Table 5:** Chromatic variables of the epicarp of cherry tomato as affected by the main factors.

Variable		Genotype			Storage Time			Storage Temperature Mean
		‘Eletta’	‘Ottymo’	‘Sugarland’	S_0_	S_7_	S_14_	
(a*/b*)^2^	T_10_	0.79	0.74	0.59	0.72	0.67	0.73	0.71 ^a^
	T_20_	0.84	0.71	0.63	0.72	0.67	0.78	0.73 ^a^
	Mean	0.81 ^a^	0.73 ^b^	0.61 ^c^	0.72 ^a^	0.67 ^b^	0.75 ^a^	
HSD_interaction_			NS			NS		
Chroma	T_10_	26.8	26.5	21.1	24.5	24.1	26.0	24.8 ^a^
	T_20_	26.1	25.2	20.3	24.3	23.1	24.1	23.8 ^b^
	Mean	26.4 ^a^	25.9 ^b^	20.7 ^c^	24.4 ^b^	23.6 ^c^	25.1 ^a^	
HSD_interaction_			NS			0.7		
TCI	T_10_	33.9	34.9	31.1	33.1	32.9	33.8	33.3 ^a^
	T_20_	34.1	34.2	31.6	33.1	32.6	34.0	33.3 ^a^
	Mean	34.0 ^a^	34.5 ^a^	31.3 ^b^	33.1 ^ab^	32.7 ^b^	33.9 ^a^	
HSD_interaction_			NS			NS		
ΔE*_ab_	T_10_	1.37	2.65	3.11	-	2.18	2.57	2.38 ^a^
	T_20_	1.49	2.36	1.77	-	1.64	2.10	1.87 ^b^
	Mean	1.43 ^b^	2.50 ^a^	2.44 ^a^	-	1.91 ^b^	2.34 ^a^	
HSD_interaction_			0.6			NS		

Different letters among factor means indicate significance at Tukey’s HSD test (*p* ≤ 0.05). Interaction values (*p* = 0.05) related to ‘storage temperature × genotype’ and ‘storage temperature × storage time’ are reported. NS: not significant; -: no data.

**Table 6:** Nutraceutical variables of cherry tomato as affected by the main factors.

Variable		Genotype			Storage Time			Storage Temperature Mean
		‘Eletta’	‘Ottymo’	‘Sugarland’	S_0_	S_7_	S_14_	
TPC (mg GAE kg^−1^ FW)	T_10_	4087	4531	4364	3997	4869	4116	4327 ^a^
	T_20_	4287	4167	3647	3982	4452	3668	4034 ^b^
	Mean	4187 ^b^	4349 ^a^	4006 ^c^	3989 ^b^	4660 ^a^	3892 ^b^	
HSD_interaction_			303			303		
Phytoene content (mg kg^−1^ FW)	T_10_	50.4	41.5	41.2	43.3	45.5	45.1	44.3 ^a^
	T_20_	53.5	38.5	41.3	43.1	41.2	48.3	44.5 ^a^
	Mean	51.9 ^a^	40.0 ^b^	41.2 ^b^	43.2 ^b^	43.3 ^b^	46.7 ^a^	
HSD_interaction_			NS			3.2		
Phytofluene content (mg kg^−1^ FW)	T_10_	42.1	38.7	32.3	39.6	37.7	35.6	37.7 ^a^
	T_20_	46.5	34.6	33.9	39.8	33.4	41.9	38.3 ^a^
	Mean	44.3 ^a^	36.6 ^b^	33.1 ^c^	39.7 ^a^	35.5 ^c^	38.8 ^b^	
HSD_interaction_			3.5			3.5		
Lycopene content (mg kg^−1^ FW)	T_10_	488	185	662	392	526	404	445 ^b^
	T_20_	556	207	701	416	577	484	488 ^a^
	Mean	552 ^b^	196 ^c^	682 ^a^	404 ^c^	552 ^a^	444 ^b^	
HSD_interaction_			35			35		
β-carotene content (mg kg^−1^ DW)	T_10_	92.0	85.2	83.4	77.0	89.7	94.0	86.9 ^a^
	T_20_	96.0	81.7	77.1	76.7	85.9	92.3	84.9 ^a^
	Mean	94.0 ^a^	83.5 ^b^	80.3 ^b^	76.8 ^c^	87.8 ^b^	93.1 ^a^	
HSD_interaction_			NS			NS		

Different letters among factor means indicate significance at Tukey’s HSD test (*p* ≤ 0.05). Interaction values (*p* = 0.05) related to ‘storage temperature × genotype’ and ‘storage temperature × storage time’ are reported. NS: not significant.
